# Circulating Extracellular Vesicles Suggest Race-Associated Transcriptomic Differences in Preterm Birth: A Pilot Study

**DOI:** 10.3390/ijms27114739

**Published:** 2026-05-25

**Authors:** Bruna Corradetti, Xiyu Ge, Kristina W. Whitworth, Elaine Symanski

**Affiliations:** 1 Center for Precision Environmental Health, Baylor College of Medicine, Houston, TX 77030, USA; xiyu.ge@bcm.edu (X.G.); kristina.whitworth@bcm.edu (K.W.W.);; 2 Department of Medicine, Section of Hematology/Oncology, Baylor College of Medicine, Houston, TX 77030, USA; 3 Department of Medicine, Section of Epidemiology and Population Sciences, Baylor College of Medicine, Houston, TX 77030, USA

**Keywords:** extracellular vesicles, plasma transcriptomics, racial disparities, preterm birth

## Abstract

Preterm birth (PTB) remains a leading cause of neonatal morbidity and mortality and disproportionately affects Black women in the United States. While racial disparities in PTB are well documented, the molecular pathways underlying these differences remain incompletely understood. Extracellular vesicles (EVs) are circulating lipid-bound particles that carry coding and non-coding RNAs reflecting cellular stress states and may serve as integrative molecular indicators of pregnancy biology. In this hypothesis-generating pilot study, EVs were isolated from maternal plasma collected at delivery from non-Hispanic Black and non-Hispanic White women with preterm and full-term births. EV concentration and size were assessed, and EV-associated mRNA and miRNA cargo were profiled by next-generation sequencing (*n* = 5 per group), enabling differential expression and pathway enrichment analyses stratified by gestational outcome. EV concentrations were significantly elevated in PTB compared with full-term deliveries (*p* < 0.0001), with a greater increase among Black participants. Analysis of EV-associated mRNA transcripts identified a shared signature enriched for platelet activation and coagulation pathways across racial groups. Race-stratified analyses revealed distinct EV miRNA profiles in PTB, with enrichment of cytokine-mediated signaling pathways among Black participants and apoptosis-related pathways among White participants, while a subset of miRNAs differed by race independent of gestational outcome. These findings support EV profiling as a framework to investigate biological pathways contributing to PTB disparities.

## 1. Introduction

Preterm birth (PTB), defined as delivery before 37 completed weeks of gestation, remains a major public health challenge, affecting approximately one in ten pregnancies in the United States (U.S.) [1] and accounting for a substantial proportion of neonatal mortality worldwide [2]. Despite advances in obstetric and neonatal care, PTB continues to represent a leading cause of infant morbidity and mortality. PTB is associated with long-lasting consequences for both offspring and mothers [3,4], including increased risks of cardiometabolic [5], neurodevelopmental [6], respiratory [7], cardiovascular [8] and mental health [9] disorders. These sequelae position PTB as a condition with enduring intergenerational health implications.

Importantly, the burden of PTB varies significantly in the U.S., with the Southern and mid-Western regions reporting higher rates of PTB as compared to other states in the nation [10]. Additionally, Black women in the U.S. experience substantially higher rates of PTB (approximately 50% greater) compared with White or Hispanic women [11], a disparity that remains largely unexplained by conventional obstetric risk factors and that continues to contribute to persistent reproductive inequities across generations [12]. These disparities highlight the need to move beyond traditional clinical risk factors and towards a more integrated understanding of the biological, environmental, and structural determinants that shape pregnancy outcomes [13,14].

Recognizing that race is a social construct, structural determinants of health (e.g., socioeconomic inequities, racial segregation, and disproportionate exposure to environmental hazards) are increasingly identified as contributors to PTB risk and its unequal distribution across populations [11,15,16,17,18]. Environmental injustice, wherein historically marginalized communities disproportionately bear the burden of toxic exposures, has emerged as a plausible driver of PTB disparities [18]. Exposure to particulate matter and other air pollutants has been linked to oxidative stress, inflammation, placental dysfunction, and endocrine disruption, pathways central to PTB pathogenesis [19]. Conversely, intergenerational socioeconomic advantage is associated with improved birth outcomes, highlighting the interplay of environmental and social determinants of reproductive health [16,20].

Advances in extracellular vesicle (EV) biology have opened new avenues to explore how environmental stressors, including both chemical and non-chemical exposures, may influence circulating molecular signals during pregnancy [21]. EVs are nanoscale, lipid-bound particles secreted by nearly all cell types that serve as natural mediators of intercellular communication [22]. They carry a diverse array of bioactive cargo (including coding and non-coding RNAs, proteins, and lipids) that reflect the physiological state of their parental cells and can modulate recipient cell behavior [23,24]. Their lipid bilayer confers protection from enzymatic degradation, making EV-associated cargo a stable and accessible source of molecular information in circulation [25]. EV levels are elevated in pathological conditions such as diabetes [26] and hypertension [27]. In addition, emerging evidence suggests that specific toxicant exposures may also alter EV abundance [28]. Through their role in intercellular communication, EVs may contribute to the propagation of environmentally induced disease processes [28,29]. Importantly, EV abundance and composition fluctuate dynamically during pregnancy and have been linked to maternal body mass index [30], inflammation [30,31], and pregnancy complications [32,33,34]. Indeed, EVs have been identified as paracrine mediators of labor, capable of trafficking from the maternal circulation to gestational tissues, where they can promote inflammatory signaling and contribute to the initiation of parturition [35].

Considering their stability and biological relevance, EVs have been leveraged as informative reporters of maternal–fetal physiology across gestation [36]. Studies comparing circulating EV protein content between spontaneous PTB and term deliveries have identified functional proteomic signatures associated with inflammation, wound healing, and coagulation pathways as early as 10–12 weeks of gestation [37]. More recently, profiling of EV-derived miRNAs among a convenience sample of 31 Brazilian pregnant women revealed distinct expression patterns implicated in PTB pathogenesis [38], supporting the relevance of EV-associated RNA cargo across populations. While these findings highlight the potential of EVs for early risk stratification, EVs collected at delivery provide a complementary window into pregnancy biology, reflecting the cumulative integration of environmental, inflammatory, and physiological exposures experienced across gestation rather than early predictive signals alone [39].

**Figure 1:**
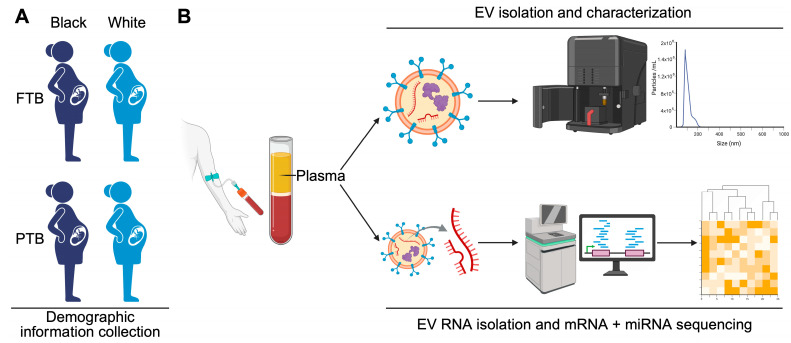
**Study design and experimental workflow.** (**A**) Participant groups included Black and White individuals with full-term birth (FTB) or preterm birth (PTB). Demographic, clinical, and exposure-related information was collected at delivery. (**B**) Maternal plasma samples were collected at delivery and used for both extracellular vesicle (EV) characterization. For each group, 10 plasma samples were analyzed; 5 were allocated for EV isolation and physicochemical characterization (i.e., size and concentration), and 5 were used for EV-derived RNA extraction followed by computational evaluation of transcriptomic signatures.

In this hypothesis-generating pilot study, designed to identify exploratory molecular patterns, we tested whether the variation in the RNA content of circulating EVs at delivery is associated with a biomolecular profile linked to gestational outcome and PTB status, and whether these molecular signatures may provide preliminary insight into biological pathways that may contribute to racial disparities in birth outcomes. To address this, we analyzed frozen plasma samples collected at delivery from a random sample of non-Hispanic Black and non-Hispanic White women with preterm and full-term pregnancies (Figure 1A) in the Maternal and Infant Environmental Health Riskscape (MIEHR) Study conducted in Houston, TX, USA. Houston, a major petrochemical hub with persistent racial disparities in PTB [18] and well-documented air quality challenges [40], provides a relevant context to investigate EVs as molecular indicators of cumulative environmental exposure and reproductive risk. EVs were evaluated for concentration, size distribution, and RNA cargo, including coding and non-coding transcripts, in relation to race and pregnancy outcome (Figure 1B). Given the limited sample size, findings are intended to be interpreted as exploratory and to inform future validation in larger cohorts.

## 2. Results

### 2.1. Participant Characteristics

Participant demographic and clinical characteristics are summarized in Table 1.

***Infant characteristics.*** Gestational age and birth weight aligned with the gestational outcome groups. Gestational age ranged from 33.6 ± 3.3 weeks in PTB-White and 34.2 ± 3.5 weeks in PTB-Black to 38.3 ± 1.5 weeks in FTB-Black and 38.4 ± 1.2 weeks in FTB-White groups. Infant birth weight showed a similar pattern, ranging from 2189 to 2477 g in the PTB groups and from 3127 to 3328 g in the FTB groups. The distribution of infant sex was similar across groups.

***Maternal characteristics.*** Data were complete except for pre-pregnancy BMI, which was missing for 50% of participants. Overall, few participants smoked or consumed alcohol during their pregnancy. However, more Black participants (PTB:70%; FTB:60%) were unmarried or living without a partner as compared to White participants (PTB:10%; FTB: 30%) and fewer Black participants were nulliparous (Black-PTB: 20%; Black-FTB: 30%; White-PTB: 50%; White-FTB: 50%). There were also differences in educational attainment (high school education or less: PTB-Black: 20%; FTB-Black: 60%; PTB-White: 10%, FTB-White: 20%) and in the neighborhoods where participants lived (ADI mean levels: PTB-Black-118.7 ± 20.3; FTB-Black: 109.4 ± 17.0 PTB-White: 89.1 ± 15.8; FTB-White: 84.7 ± 11.6 in FTB-White). In terms of delivery mode, a greater percentage of Black participants had cesarean delivery (PTB-Black: 70%; FTB-Black: 40%) than White participants (PTB-White: 20%; FTB-White: 30%).

### 2.2. Circulating EV Concentrations Are Elevated in White Participants and Participants with PTB

**Figure 2:**
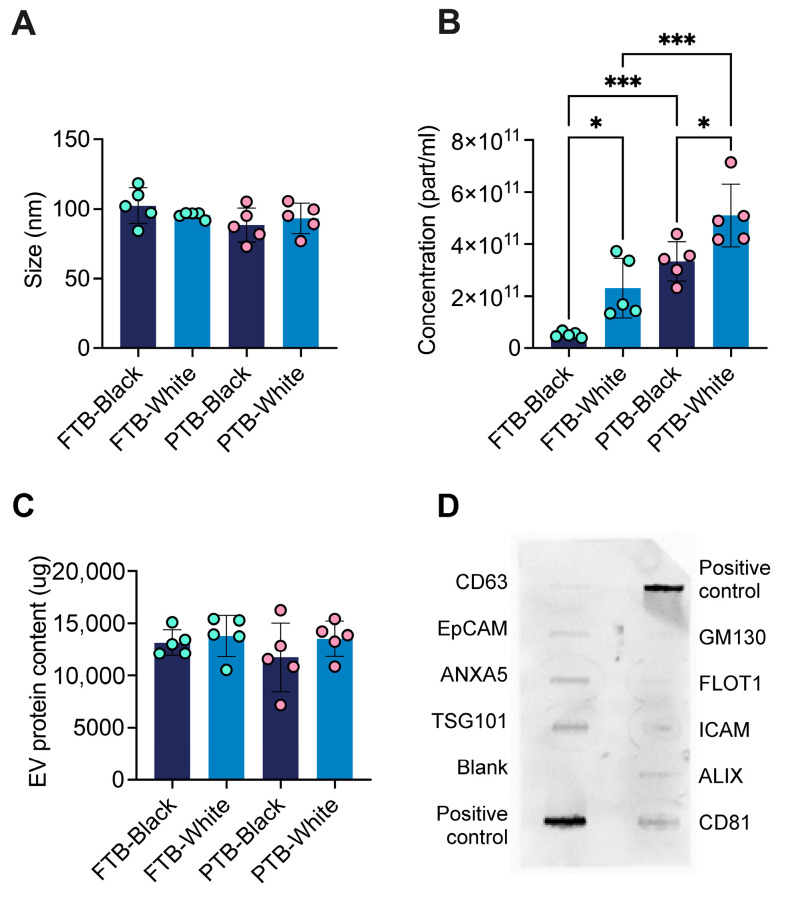
**EV physicochemical characterization across racial and gestational outcome groups.** (**A**) Mean EV diameter, (**B**) EV concentration, (**C**) total EV protein content measured in maternal plasma from Black and White participants with full-term birth (FTB) or preterm birth (PTB), and (**D**) EV protein marker profiling using the Exo-Check Exosome Antibody Array. Representative array blots demonstrate expression of established EV-associated markers, including CD63, CD81, ALIX, FLOT1, ICAM1, EpCAM, ANXA5, and TSG101, with minimal expression of GM130, a marker associated with cellular contamination. Bars represent mean ± SD for each group, with individual data points overlaid. Statistical comparisons were performed using one-way ANOVA followed by Tukey’s multiple comparisons test (* *p* < 0.05; *** *p* < 0.001).

EVs isolated from maternal plasma across all groups fell within the expected exosomal size range, with no significant differences in mean diameter among groups (Figure 2A). Mean EV diameters were 102 ± 12 nm for FTB-Black, 95 ± 2 nm for PTB-Black, 88 ± 11 nm for FTB-White, and 93 ± 10 nm for PTB-White. In contrast, maternal plasma EV concentrations differed significantly across groups (Figure 2B). Participants with PTB exhibited higher EV concentrations than those with full-term deliveries (*p* < 0.0001). This increase appeared more pronounced among Black participants, in which EV concentrations were elevated approximately 6.5-fold in PTB compared to FTB (3.4 × 10^11^ ± 7.0 × 10^10^ vs. 5.1 × 10^10^ ± 1.0 × 10^9^ particles/mL). Among White participants, EV concentrations were approximately twofold higher in PTB compared with FTB (5.1 × 10^11^ ± 1.0 × 10^11^ vs. 2.3 × 10^11^ ± 1.0 × 10^11^ particles/mL). Racial comparisons showed that both FTB-Black and PTB-Black participants exhibited significantly lower EV concentrations than their White counterparts (*p* < 0.05). Total EV protein content did not differ significantly among groups (Figure 2C). EV protein components were profiled, and a representative array blot is shown in Figure 2D. The analysis demonstrated the expression of established EV-associated markers, including CD63, CD81, ALIX, FLOT1, ICAM1, EpCAM, ANXA5, and TSG101, with minimal expression of GM130, a marker associated with cellular contamination. These findings further support the identity and relative purity of the isolated EV preparations.

### 2.3. Enrichment of Coagulation-Related Pathways Is Observed as a Shared PTB-Associated Feature Across Racial Groups

Sequencing library quality control (QC) metrics are summarized in Table 2. With RNA input amounts ranging from 0.2 to 2.9 ng across 20 samples, 20 mRNA and 20 miRNA libraries were successfully generated. For mRNA libraries, an average of 30–112 million paired-end reads per sample was obtained, with mean GC content ranging from 45% to 52% and alignment rates to the hg38 reference genome ranging from 64% to 88%. After filtering, 48,517 unique transcripts were detected across all samples. For miRNA libraries, an average of 16–30 million paired-end reads per sample was generated, yielding 578 unique miRNAs across all samples. Volcano plots summarizing differentially expressed mRNAs and miRNAs are shown in Supplementary Figure S1.

**Figure 3:**
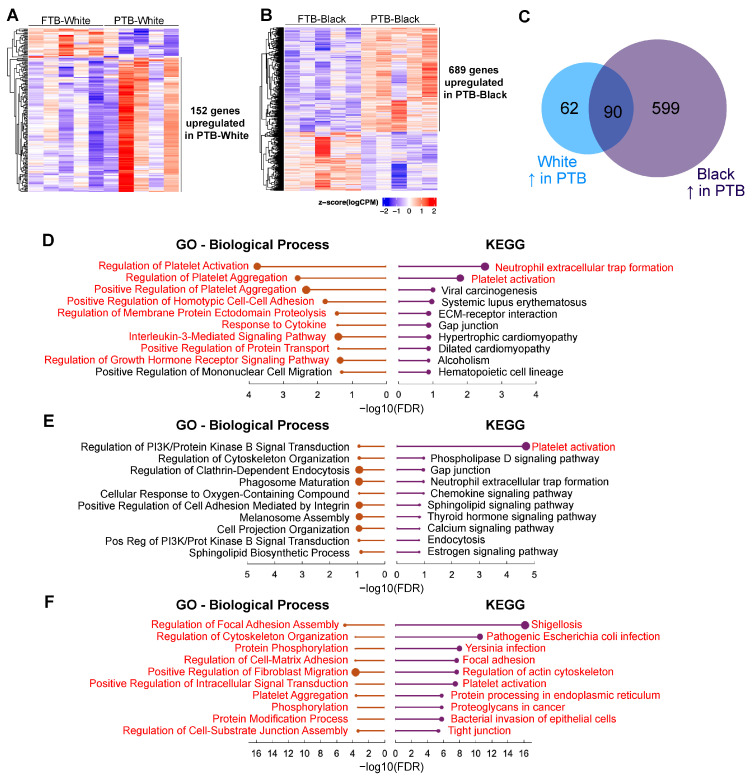
**Gestation-associated EV mRNA signatures in Black and White participants.** (**A**) Heatmap of differentially expressed EV-associated mRNAs between full term birth (FTB) and preterm birth (PTB) among White participants (PTB-White vs. FTB-White). (**B**) Heatmap of differentially expressed EV-associated mRNAs between FTB and PTB among Black participants (PTB-Black vs. FTB-Black). (**C**) Venn diagrams illustrating overlap between Black and White participants for EV-mRNAs upregulated in PTB. Up arrow: upregulated. (**D**) Enriched Gene Ontology (GO) Biological Process terms and KEGG pathways derived from EV-mRNAs consistently upregulated in PTB across both White and Black participants. (**E**) Enriched GO Biological Process terms and KEGG pathways derived from EV-mRNAs uniquely upregulated in PTB-White participants. (**F**) Enriched GO Biological Process terms and KEGG pathways derived from EV-mRNAs uniquely upregulated in PTB-Black participants. Significantly enriched GO and pathways are highlighted in red (FDR < 0.05). Lollipop plots display pathway significance (−log10 FDR) with combined scores indicating dot size.

Among White participants (PTB-White vs. FTB-White), 185 differentially expressed mRNAs were identified, including 152 upregulated and 33 downregulated in PTB (Figure 3A). Among Black participants (PTB-Black vs. FTB-Black), 1078 differentially expressed mRNAs were identified (689 upregulated, 389 downregulated) (Figure 3B). Comparison of these gene lists revealed 93 overlapping mRNAs differentially expressed between PTB and FTB across racial groups, of which 90 were consistently upregulated (Figure 3C) and 3 were downregulated in PTB (*NT5C3B*, *ZSWIM3* and *LGALS9C*). These shared transcripts point towards a potentially conserved PTB-associated EV mRNA pattern across racial groups.

Functional enrichment analysis of the 90 genes consistently upregulated in PTB across racial groups revealed enrichment of two KEGG pathways: platelet activation and neutrophil extracellular trap (NET) formation. Among the nine significantly enriched GO Biological Process terms, the top three were related to coagulation processes, including regulation of platelet activation, regulation of platelet aggregation, and positive regulation of platelet aggregation (Figure 3D). Full enrichment results are provided in Supplementary File S2. Consistent with these race-stratified observations, PTB vs. FTB comparisons performed across the entire study population showed similar transcriptional patterns, including enrichment of coagulation-related pathways at the mRNA level (Supplementary Figure S2A,B) and GO Biological Process terms associated with cellular signaling and apoptotic regulation among miRNA target genes (Supplementary Figure S2C,D).

To provide orthogonal validation of selected EV RNA-seq findings, five differentially expressed transcripts (*ENDOD1*, *TIMP1*, *ITGA2B*, *F2R*, and *FCER1G*) were evaluated by qRT-PCR. Among these candidates, *TIMP1* was reliably detected and confirmed to be increased in PTB samples compared with FTB controls, consistent with the RNA-seq results (Supplementary Figure S3). The remaining transcripts showed inconsistent amplification or levels below the reliable detection threshold, likely reflecting the low abundance and fragmented nature of EV-associated RNA cargo (Supplementary Table S1).

### 2.4. EV mRNA and miRNA Signatures Suggest Additional Race-PTB-Associated Mechanisms

Race-stratified analyses identified distinct molecular patterns associated with PTB. In PTB-White participants, 62 uniquely upregulated genes were identified and showed enrichment for platelet activation-related pathways (Figure 3E). In contrast, among the 599 uniquely upregulated genes in PTB-Black participants, platelet aggregation and activation remained among the top enriched GO and KEGG categories, while additional enriched KEGG pathways were primarily related to infectious disease and immune-associated processes. The top GO Biological Process terms in the PTB-Black group included processes related to cell adhesion, cytoskeletal organization, and protein modification (Figure 3F).

Analysis of genes downregulated in PTB identified only one significantly enriched KEGG pathway (glycine, serine, and threonine metabolism) in the PTB-Black group, whereas no significantly enriched biological processes were observed among downregulated genes in PTB-White participants. Full enrichment results are provided in Supplementary File S1.

**Figure 4:**
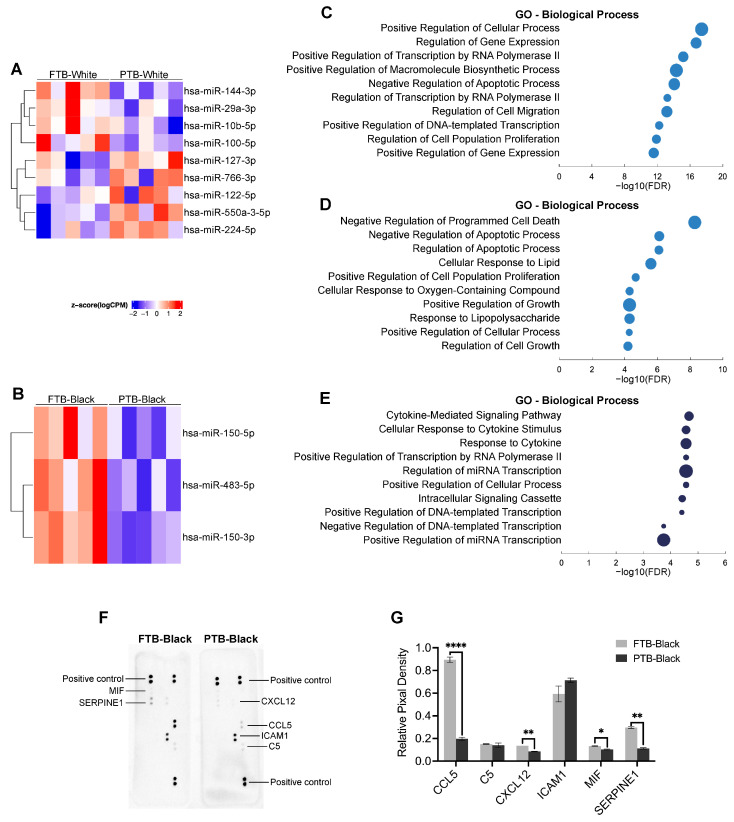
**Gestation-associated EV miRNA signatures in Black and White participants.** (**A**) Heatmap of differentially expressed EV-associated miRNAs between full-term birth (FTB) and preterm birth (PTB) among White participants (PTB-White vs. FTB-White). (**B**) Heatmap of differentially expressed EV-associated miRNAs between FTB and PTB among Black participants (PTB-Black vs. FTB-Black). (**C**) Enriched Gene Ontology (GO) Biological Process terms derived from experientially validated target genes of EV-miRNAs downregulated among White participants with PTB. (**D**) Enriched GO Biological Process terms derived from experimentally validated target genes of EV-miRNAs upregulated in PTB among White participants. (**E**) Enriched GO Biological Process terms derived from experientially validated target genes of miRNAs downregulated in PTB among Black participants. Enrichment analyses were performed using Enrichr. Bubble plots display pathway significance (−log10 FDR) and combined scores (dot size). (**F**) Representative cytokine array blot of serum samples from FTB-Black and PTB-Black participants. (**G**) Quantification of selected cytokines detected by cytokine array analysis. Cytokine profiling was performed to provide preliminary biological support for the cytokine-mediated signaling pathways identified through EV-miRNA target enrichment analyses. Statistical comparisons were performed using Student’s *t* test (* *p* < 0.05; ** *p* < 0.01; **** *p* < 0.0001).

At the miRNA level, nine differentially expressed miRNAs were identified in the White cohort, including four downregulated (hsa-miR-144-3p, hsa-miR-29a-3p, hsa-miR-10b-5p and hsa-miR-100-5p) and five upregulated (hsa-miR-127-3p, hsa-miR-766-3p, hsa-miR-122-5p, hsa-miR-550a-3-5p and hsa-miR-224-5p) in PTB compared with FTB (Figure 4A). Among Black participants, three differentially expressed miRNAs (hsa-miR-150-5p, hsa-miR-483-5p and hsa-miR-150-3p) were identified, all downregulated in PTB (Figure 4B).

Functional enrichment of experimentally validated target genes suggested that miRNAs dysregulated in PTB among White participants were associated with pathways related to gene expression regulation, cellular proliferation, and apoptosis (Figure 4C,D). In contrast, target genes of the three miRNAs downregulated in PTB among Black participants showed enrichment for cytokine-mediated signaling pathways, which appeared to be the most significantly enriched pathways (Figure 4E). To provide preliminary biological support for these observations, cytokine array analysis of serum samples from FTB-Black and PTB-Black participants revealed altered expression of CCL5, CXCL12, MIF and SERPINE1 in PTB-Black compared to FTB-Black (Figure 4F,G). The full list of miRNA targets and enrichment results are provided in Supplementary File S2.

### 2.5. EV miRNA Signatures Identify Race-Associated Molecular Features Independent of Gestational Outcome

**Figure 5:**
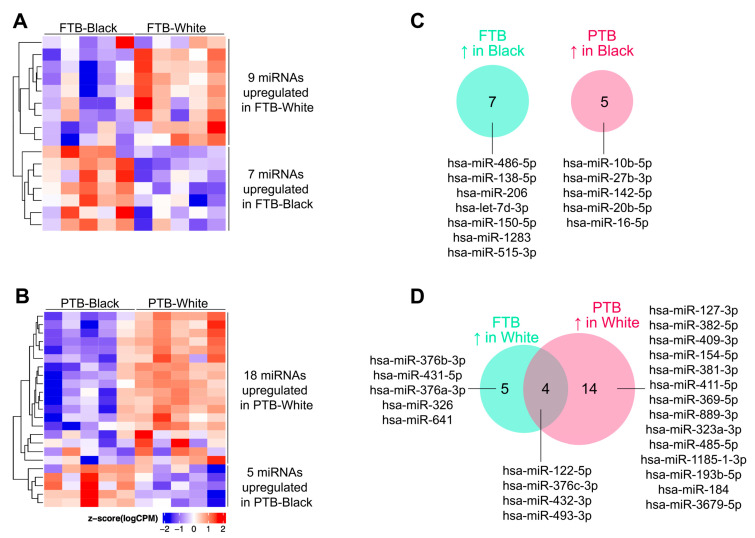
**Race-associated EV miRNA signatures among participants with full-term birth (FTB) and preterm birth (PTB).** (**A**) Heatmap of differentially expressed EV-associated miRNAs between Black and White participants with FTB (FTB-Black vs. FTB-White). (**B**) Heatmap of differentially expressed EV-associated miRNAs between Black and White participants with PTB (PTB-Black vs. PTB-White). (**C**) Venn diagram illustrating overlap between participants with FTB and PTB for miRNAs upregulated in Black participants. Up arrow: upregulated. (**D**) Venn diagram illustrating overlap between participants with FTB and PTB for EV-miRNAs upregulated in White participants. Up arrow: upregulated.

Among participants with FTB (FTB-White vs. FTB-Black), 16 differentially expressed miRNAs were identified, including 7 upregulated in Black participants and 9 upregulated in White participants (Figure 5A). For women whose infants were preterm (PTB-White vs. PTB-Black), 23 differentially expressed miRNAs were identified, with 5 upregulated in Black participants and 18 upregulated in White participants (Figure 5B). No miRNAs were consistently upregulated in Black participants across both gestational outcome groups (Figure 5C). In contrast, 4 miRNAs (hsa-miR-122-5p, hsa-miR-376c-3p, hsa-miR-432-3p, and hsa-miR-493-3p) were consistently upregulated in White participants regardless of gestational outcome (Figure 5D). These miRNAs may represent a preliminary race-associated EV miRNA pattern independent of PTB status, warranting further validation in a larger cohort.

## 3. Discussion

**Figure 6:**
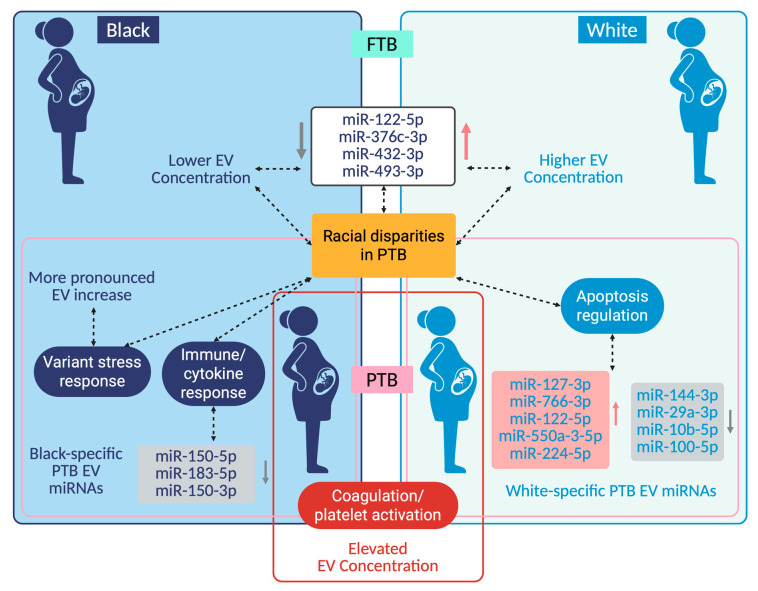
Conceptual model summarizing hypothesized EV signatures in preterm birth (PTB) and racial differences. Schematic summary of the study findings integrating differences in extracellular vesicle (EV) concentration and RNA cargo across racial groups and gestational outcomes. Preterm birth (PTB) is characterized by increased EV concentration and enrichment of coagulation-related pathways, including platelet activation. Race-stratified analyses highlight distinct EV-associated miRNA signatures, with cytokine- and immune-related signaling enriched among Black participants and apoptosis-related pathways enriched among White participants. Together, these findings are consistent with EV abundance and RNA cargo reflecting biological processes linked to PTB and may provide insight into mechanisms contributing to disparities in pregnancy outcomes. This model is presented as a hypothesis-generating framework and does not represent a demonstrated mechanism. Up arrow: upregulated. Down arrow: downregulated.

In this study, we confirmed the identity and purity of EVs isolated from maternal plasma collected at delivery, and showed that they capture molecular signatures associated with gestational outcome and maternal race (Figure 6). When integrating physicochemical characterization and transcriptomic profiling, EV concentration and RNA cargo are consistent with late-gestation biological states in PTB and encompass both shared and potentially race-associated molecular patterns. These EV profiles may provide a molecular readout of systemic stressors accumulated across pregnancy. Circulating EVs are increasingly recognized as dynamic components of maternal–fetal communication, with abundance rising across gestation and reported elevations in pregnancy complications, such as preeclampsia and GDM [41,42,43].

Consistent with this literature, EV concentrations were elevated at delivery in preterm pregnancies, a pattern consistent with a heightened systemic stress response [44,45]. Rather than representing a predisposing factor, this increase likely reflects the cumulative activation of inflammatory [44], metabolic [44], and vascular [27] pathways accompanying pathological parturition. EV-associated cargo mirrors cellular stress states [44,46], and EVs may contribute to signal propagation at the maternal–fetal interface through transfer of pro-inflammatory and pro-coagulant cargo [47,48], modulation of endothelial permeability [49], and immune cell activation [50], although functional mediation cannot be inferred from the present data.

The temporal context of our sampling at delivery [51] captures a terminal phase of pregnancy biology, encompassing exposures and physiological adaptations accrued across gestation [39]. Accordingly, elevated EV concentrations in PTB are best interpreted as downstream manifestations of labor-associated inflammatory and vascular stress [52,53], rather than early predictors of an adverse outcome. This interpretation aligns with prior reports demonstrating dynamic EV fluctuations in response to inflammatory cues and advancing gestation [52]. Conversely, earlier gestational studies have reported reduced EV concentrations in pregnancies that later culminated in PTB [36], indicating that dysregulation of EV biogenesis, release, or clearance may evolve across pregnancy [54,55]. Together, these observations support a stage-dependent model of EV dynamics [56]. Notably, although EV concentrations were elevated in PTB across both racial groups, the difference was greatest among Black participants. Such differences likely reflect variation in cumulative inflammatory and vascular stress across pregnancy [52,53]. As race is a social rather than biological construct, these findings are interpreted as reflecting differential lived exposures rather than intrinsic biological variation [57].

Within this context, race-specific EV miRNA signatures observed in PTB highlight divergence in stress-response pathways across populations. Several miRNAs differentially expressed between PTB-Black and PTB-White participants have been linked to inflammatory signaling (hsa-miR-382-5p, hsa-miR-10b-5p, hsa-miR-27b-3p) [58,59,60], metabolic regulation (hsa-miR-193b-5p) [61], and oxidative stress responses (hsa-miR-184, hsa-miR-10b-5p, hsa-miR-27b-3p) [59,62,63]. Rather than implicating individual miRNAs as causal drivers, these patterns are consistent with coordinated modulation of EV cargo in the context of late-gestation stress adaptation [64]. Such signatures represent molecular indicators of differential biological responses to labor-associated stress, potentially contributing to the amplified EV surge observed among Black participants.

An additional observation was racial differences in EV abundance independent of gestational outcome, with White participants exhibiting higher circulating EV concentrations than Black participants in both PTB and FTB groups. This pattern is consistent with previously reported racial difference in plasma EV concentration and points to differences in EV regulatory dynamics, potentially involving vesicle biogenesis, release, uptake, or immune-mediated clearance [65], that are not solely attributable to pregnancy complications. Notably, these differences coincided with consistent differential expression of four EV-associated miRNAs (hsa-miR-122-5p, hsa-miR-376c-3p, hsa-miR-432-5p and hsa-miR-493-3p) between Black and White participants regardless of gestational outcome, indicating a race-associated EV molecular profile present across pregnancy states. As above, these differences should be interpreted within a framework in which race serves as a proxy for cumulative social, environmental, and social exposures that may shape EV regulatory dynamics. Thus, observed race-associated variation in EV abundance and cargo likely reflects differential lived experiences and biological responses rather than intrinsic racial biology. The known biological functions of these miRNAs implicate metabolic regulation, placental and epigenetic stress responses, and cellular stress adaptation. Specifically, hsa-miR-122-5p regulates systemic metabolism, inflammation, and oxidative stress [66], while hsa-miR-376c-3p and hsa-miR-432-5p belong to an imprinted placental miRNA cluster linked to developmental and stress-responsive regulation [67]. hsa-miR-493-3p has been associated with melanocyte stress responses and apoptotic regulation [68]. Collectively, this miRNA profile indicates differences in EV homeostasis that may influence baseline vesicle abundance and responsiveness to gestational stressors. These molecular patterns occurred among Black participants who lived in more disadvantaged neighborhoods as compared with White participants and provide contextual insight into potential area-level differences in social and environmental stress exposures that warrant investigation.

Exploratory trends highlight the importance of integrating exposure metrics with EV profiling in larger cohorts to contextualize stress-responsive vesicle biology [69,70]. Analysis of EV-associated RNA cargo reveals a layered molecular organization in PTB, in which regulatory signals captured by EV miRNAs are integrated into downstream effector pathways reflected at the mRNA level. Transcriptomic profiling demonstrated enrichment of mRNAs involved in platelet activation and coagulation pathways in PTB, independent of maternal race. These data support a model in which PTB may involve common terminal biological programs, most prominently coagulation and platelet activation, while upstream regulatory programs diverge across racial groups. This aligns with established links between PTB and hypercoagulability [48,71], while extending this concept to the EV compartment. The prominence of coagulation-related transcripts indicates a conserved biological response to pathological parturition, potentially arising from placental microvascular injury, endothelial activation, or oxidative stress [49,72]. Given the role of platelet-derived EVs in vascular remodeling and inflammation [73], EVs are best interpreted as carriers of thrombo-inflammatory signals characteristic of PTB rather than primary disease initiators.

Within this shared downstream framework, race-stratified analyses reveal divergence at the regulatory level, reflected primarily in EV-associated miRNA signatures. EV mRNA cargo from Black participants with PTB exhibited additional enrichment of immune and infection-related pathways, alongside miRNA signatures (has-miR-150-3p, has-miR-150-5p, has-miR-483-3p) whose validated targets were enriched for cytokine-mediated signaling processes, consistent with heightened or sustained immune activation [74,75,76]. In contrast, PTB-associated regulatory signatures in White participants were characterized by a more restricted mRNA profile, with miRNA target gene enrichment pointing toward apoptosis and cell survival pathways. A literature review indicated that six of these nine miRNAs have previously been implicated in the regulation of apoptotic processes (hsa-miR-127-3p [77], hsa-miR-122-5p [78], hsa-miR-144-3p [79], hsa-miR-29a-3p [80], hsa-miR-10b-5p [81], hsa-miR-100-5p [82]), with two reported as EV-associated cargos (hsa-miR-122-5p [83] and hsa-miR-100-5p [84]). The relatively small number of differentially expressed miRNAs identified in race-stratified comparisons, particularly in the Black PTB group, may limit the stability of downstream pathway enrichment analyses. Accordingly, these results should be interpreted with caution and considered exploratory.

Overall, these data support a conceptual, hypothesis-driven framework in which PTB may converge on shared downstream effector pathways, while regulatory programs encoded in EV miRNAs may differ across populations. However, this study has several limitations that should be considered when interpreting the findings. First, the sample size was limited (*n* = 5 per group for transcriptomic analyses), which constrains statistical power and precludes definitive conclusions, particularly for race-stratified comparisons and interaction effects between race, environmental exposures, and molecular features. As such, the results should be interpreted as exploratory and hypothesis-generating. Second, functional inferences are primarily based on computational pathway enrichment analyses and, although biologically consistent with prior literature, require further experimental validation to confirm mechanistic relevance.

To partially address this, selected EV-associated transcripts identified by RNA sequencing were evaluated by qRT-PCR, and cytokine profiling was performed to provide preliminary biological support for the cytokine-mediated signaling pathways identified through EV-miRNA target enrichment analyses. TIMP1 expression was confirmed to be increased in PTB samples, supporting the directionality of the sequencing findings, while cytokine array analysis revealed altered expression of several inflammatory mediators in PTB-Black participants compared with FTB-Black participants. However, broader validation of EV-associated transcripts remained technically challenging because EV-associated RNAs are often present at low abundance and in fragmented forms [85], which may limit amplification efficiency using conventional qRT-PCR approaches. Third, EVs were collected at delivery, capturing a late-stage snapshot of pregnancy biology and the acute physiology of parturition; therefore, these findings reflect cumulative physiological and environmental influences rather than early predictive biomarkers of PTB. Fourth, while this study focused primarily on EV-associated RNA cargo, EVs carry a heterogeneous molecular composition, including proteins and lipids [22], that was only partially explored here and may provide additional layers of biological insight. Finally, because of the small sample size, a limitation of the present investigation was that a host of individual-level factors that are known to influence EV profiles [30,86] (e.g., BMI, parity, infection status, delivery mode), or neighborhood features where participants lived could not be evaluated as potential confounders of the racial differences that were observed. Thus, future work integrating longitudinal sampling, multi-omic profiling across EV cargo classes, and detailed individual- and neighborhood-level exposure data in larger, diverse cohorts will be essential to validate these findings and further elucidate the role of EVs in PTB biology.

## 4. Materials and Methods

### 4.1. Sample and Demographic Information Collection

This investigation relied on deidentified data and biobanked plasma samples for a subset of women who participated in the MIEHR Study that has been described elsewhere [51]. Briefly, non-Hispanic Black and non-Hispanic White (hereafter referred to as ‘Black’ and ‘White’) women were recruited at delivery from three large obstetric hospitals in the Texas Medical Center in Houston, TX, USA. After enrollment, field staff administered questionnaires and abstracted data from participants’ electronic health records (Figure 1). Infant data included gestational age (weeks) at delivery, birth weight (g), and sex. Available maternal variables that were used in the present investigation included age (years), race (Black or White), education (high school or less, some college or higher), marital or partnership status (married/living with a partner, single/separated/widowed/divorced), parity (0, 1, 2, or 3+), pre-pregnancy body mass index (BMI) (kg/m^2^), and cigarette smoking and alcohol use during pregnancy. Information on participant residential address at delivery was also obtained and later geocoded using ArcGIS Pro (version 3.1, ESRI, Redlands, CA) based on the 2020 U.S. Census. The Area Deprivation Index (ADI), a composite measure of neighborhood deprivation that relies on census-tract level information about education, employment, income, and poverty [87], was constructed for the study area using data from the 2022 U.S. Census American Community Survey (ACS) and the appropriate metric constructed [88] linked to each mother’s geocoded address.

As part of the MIEHR protocol, blood samples were collected shortly after delivery (within 24–48 h) and transported within one hour of collection for biospecimen processing where they were centrifuged, aliquoted and stored at −80 °C. For the present study, deidentified plasma samples and associated data were obtained for 40 participants randomly sampled into four groups: Black women with PTB (PTB-Black, *n* = 10), Black women with full-term birth (FTB) (FTB-Black, *n* = 10), White women with PTB (PTB-White, *n* =10), and White women with FTB (FTB-White, *n* = 10). PTB cases included both spontaneous and medically indicated preterm births, with each PTB group comprising 70% spontaneous and 30% medically indicated cases. Within each group, a subset of five samples (*n* = 5 per group) were selected for EV characterization, and the remaining samples were used for EV transcriptomic profiling, including the analysis of EV-associated miRNAs and mRNAs (*n* = 5 per group).

### 4.2. EV Isolation and Physiochemical and Molecular Characterization

EVs were isolated from 500 µL maternal plasma samples (*n* = 5 per group) using the ExoEasy Kit (Qiagen, Germantown, MD, USA) and characterized according to the Minimal Information for Studies of Extracellular Vesicles (MISEV) guidelines established by the International Society for Extracellular Vesicles [89]. Particle size distribution and concentration were determined by nanoparticle tracking analysis (NTA; NanoSight NS300, Malvern, Westborough, MA, USA), with the instrument calibrated using 100 nm polystyrene beads prior to each run. For each sample, five 60-s videos were acquired, with a detection threshold set at 5 and analyzed in triplicate. Total EV protein concentration was quantified using the Pierce BCA Protein Assay Kit (Thermo Fisher Scientific, Waltham, MA, USA). For EV protein characterization, 50 µg of EV protein per sample was analyzed using the Exo-Check Exosome Antibody Array (System Biosciences, Palo Alto, CA, USA) according to the manufacturer’s instructions.

### 4.3. EV RNA Profiling and Bioinformatic Analysis

**RNA isolation.** Plasma samples (500 µL; *n* = 5 per group) were processed for isolation of total RNA, including miRNAs, using the ExoRNeasy Midi Kit (Qiagen, Germantown, MD, USA). During extraction, a pool of 52 QIAseq miRNA QC Spike-Ins (Qiagen, Germantown, MD, USA) was added to each sample to assess RNA quality, reproducibility, and potential contamination from enzymatic inhibitors or nucleases. Spike-in controls were additionally used to assess hemolysis, enabling the identification of samples with evidence of plasma miRNA contamination; no samples met exclusion criteria based on hemolysis.

**Library preparation and sequencing.** RNA samples extracted from EVs were processed at the Genomic and RNA Profiling Core at BCM for RNA quality control, and Next-Generation Sequencing (NGS). Library preparation was performed using the QIAseq miRNA Library Kit (Qiagen, Germantown, MD, USA) for miRNA profiling and SMARTer^®^ Stranded Total RNA-Seq Kit v3 (Takara Bio, San Jose, CA, USA) with ribodepletion for mRNA profiling, followed by sequencing on the Illumina NovaSeq 6000 platform. Paired-end sequencing (150 bp) was performed, and FASTQ files were generated for downstream analysis.

**Bioinformatic analysis.** Post-sequencing quality control and bioinformatic analyses were conducted by the Multi-Omics Bioinformatics Core at the Advanced Technology Core at BCM. Paired-end reads were trimmed using TrimGalore and mapped to the hg38 genome build using STAR. For mRNA reads, aligned reads were quantified against GENECODE gene model annotations [90] using FeatureCounts [91]. After removal of unwanted variation (RUVr) [92] and upper-quartile normalization, differential expression analysis was performed using the edgeR likelihood ratio test (LRT) [93]. For miRNA reads, only miRNAs expressed at >1 count per million in at least three samples were included in differential analysis. Given the exploratory nature of this pilot study, mRNAs and miRNAs were considered significantly differentially expressed when exhibiting a fold change > 1.25 and a false discovery rate (FDR)-adjusted *p*-value < 0.25. Given the pilot nature of this study and the limited sample size, these thresholds were selected to increase sensitivity for detecting potential signals, with the understanding that this approach may also increase the risk of false positives. Accordingly, identified features are interpreted as exploratory and intended to guide hypothesis generation rather than define robust molecular signatures.

### 4.4. qRT-PCR Validation

Selected differentially expressed EV-associated transcripts identified by RNA sequencing were evaluated by quantitative reverse transcription PCR (qRT-PCR) for orthogonal validation. Candidate genes included ENDOD1, TIMP1, ITGA2B, F2R, and FCER1G, selected based on differential expression patterns associated with PTB across racial groups. Total EV RNA was isolated using the exoRNeasy Midi Kit (QIAGEN) and quantified on a 2100 Bioanalyzer RNA Pico Chip (Agilent Technologies, Santa Clara, CA, USA), followed by reverse transcription into cDNA using a High-Capacity cDNA Reverse Transcription Kit (Applied Biosystems, Carlsbad, CA, USA). qPCR was performed using the probes of selected genes and TaqMan™ Fast Advanced Master Mix (Thermo Fisher Scientific, Waltham, MA, USA) according to manufacturer protocols. Relative transcript expression compared to housekeeping gene *ACTB* was assessed between PTB and FTB groups using the 2(-Delta Delta C(T)) method [94].

### 4.5. Functional Analysis of Differentially Expressed mRNAs and miRNAs

**mRNA analysis.** Gene ontology (GO) enrichment and Kyoto Encyclopedia of Genes and Genomes (KEGG) pathway analyses [95,96] were performed using Enrichr [97,98,99]. Input gene lists were derived from the differential expression analyses using the same significance thresholds described above. Transcriptional signatures associated with PTB were evaluated in the overall cohort and further analyzed within each racial group to enable a comparison of shared and race-specific transcriptional responses. Resulting gene sets were submitted to Enrichr for functional annotation within the GO-Biological Process category and KEGG pathways. Enrichment significance was determined using Enrichr’s FDR correction, and results included fold enrichment, gene counts, and −log10(FDR). GO terms and KEGG signaling pathways were considered significantly enriched at FDR < 0.05.

**miRNA analysis.** The same comparative framework applied to mRNAs was used for miRNAs, with identical significance thresholds. For miRNAs differentially expressed between PTB and FTB in each racial group (PTB-Black vs. FTB-Black; PTB-White vs. FTB-White), experimentally validated target genes were identified using miRTargetLink 2.0 [100] followed by GO-Biological Process enrichment analysis using Enrichr. Given the smaller number of differentially expressed miRNAs, individual miRNAs were additionally examined, and analyses were structured to assess both shared and group-specific features across gestational and racial groups. In addition, PTB vs. FTB comparisons across the entire study population were performed to identify race-independent transcriptional signatures for both mRNAs and miRNAs.

### 4.6. Cytokine Array Analysis

Serum cytokine profiling was performed using the Proteome Profiler Human Cytokine Array Kit (R&D Systems, Minneapolis, MN, USA) according to the manufacturer’s instructions. Briefly, 200 µL of serum sample was used per membrane to evaluate the relative expression of 36 human cytokines and chemokines. Membranes were imaged, and spot pixel density was quantified using ImageJ software (Version 1.53). Signal intensity for each cytokine was normalized to the positive control spots present on each membrane to generate relative mean pixel density values for downstream comparative analyses between groups.

### 4.7. Statistical Analysis

Participant characteristics were summarized by study group (FTB-Black, PTB-Black, FTB-White and PTB-White). Continuous variables (i.e., maternal age, BMI, gestational age at delivery, infant birth weight, and ADI) were reported as means ± standard deviations (SD). Categorical variables (e.g., infant sex, education, marital status, parity, smoking and alcohol use during pregnancy) were summarized as counts and percentages.

## 5. Conclusions

In conclusion, this study shows that EVs isolated from maternal plasma at delivery capture molecular signatures related to gestational outcome and maternal race, reflecting cumulative biological stress across pregnancy. Although plasma samples were collected at delivery and therefore capture late-stage pregnancy biology and the acute physiology of parturition rather than early predictive events, the observed EV-associated RNA signatures highlight regulatory and effector pathways relevant to PTB and associated health disparities.

Given the growing interest in miRNAs as predictive biomarkers of PTB, our findings provide context for interpreting EV-associated miRNA signals within the broader literature. Numerous prior studies have identified altered plasma, serum, or placental miRNAs associated with PTB that overlap with our PTB-associated miRNAs, including hsa-miR-150-5p [101], hsa-miR-122-5p [102], hsa-miR-127-3p [103] and hsa-miR-766-3p [104]. However, most prior investigations evaluated total circulating or cell-free miRNAs, and relatively few focused specifically on miRNAs packaged within EVs [38,104]. Compared with cell-free nucleic acids, EV-encapsulated RNA offers stability, reflects cell-to-cell origin-specific biology, and integrates intercellular signaling across tissues [25], supporting its utility as a complementary approach for studying pregnancy biology. Additionally, relatively few studies examining miRNAs in PTB have incorporated race-stratified analyses [18,45,47], representing a critical gap given persistent inequity in PTB. By characterizing EV-associated miRNAs within a racially stratified study population, this study provides initial evidence that molecular signaling pathways linked to PTB may vary across populations. However, no causal or race-associated effects can be inferred from the findings, and this pilot study instead provides hypothesis-generating results. Overall, the data support the use of EVs as integrative molecular readouts of material adaptation to cumulative stress across pregnancy, rather than validated biomarkers of disease. By capturing biologically encoded signals at the intersection of gestational outcome, race, and systemic stress, EV profiling offers a framework for future longitudinal studies aimed at understanding PTB biology and informing more precise and equitable strategies for risk assessment and intervention.

## Figures and Tables

**Table 1:** **Sociodemographic and clinical characteristics of participants (*n* = 40) by gestational outcome and racial group.** Continuous variables are presented as mean ± standard deviation (SD), and categorical variables are presented as counts (%). Percentages were calculated based on available data.

	PTB-Black (*n* = 10)	FTB-Black (*n* = 10)	PTB-White (*n* = 10)	FTB-White (*n* = 10)
**Infant characteristics**				
**Gestational age (weeks)**	34.2 ± 3.5	38.3 ± 1.5	33.6 ± 3.3	38.4 ± 1.2
**Birth weight (g)**	2477 ± 741.1	3127 ± 519.4	2189 ± 856.2	3328 ± 411.4
**Sex (*n*, %)**				
Female	4 (40%)	7 (70%)	5 (50%)	7 (70%)
Male	6 (60%)	3 (30%)	5 (50%)	3 (30%)
**Maternal characteristics**				
**Maternal age (y)**	28.0 ± 5.9	28.6 ± 7.2	28.8 ± 6.1	30.4 ± 7.0
BMI (kg/m^2^) *	32.7 ± 11.6	41.0 ± 15.1	33.7 ± 8.4	25.5 ± 5.5
**Area Deprivation Index**	118.7 ± 20.3	109.4 ± 17.0	89.1 ± 15.8	84.7 ± 11.6
**Education, *n* (%)**				
≤High school	2 (20%)	6 (60%)	1 (10%)	2 (20%)
Some college or higher	8 (80%)	4 (40%)	9 (90%)	8 (80%)
**Smoking in pregnancy, *n* (%)**				
No	8 (80%)	9 (90%)	10 (100%)	10 (100%)
Yes	2 (20%)	1 (10%)	0 (0%)	0 (0%)
**Alcohol in pregnancy, *n* (%)**				
No	9 (90%)	8 (80%)	9 (90%)	8 (80%)
Yes	1 (10%)	2 (20%)	1 (10%)	2 (20%)
**Parity, *n* (%)**				
0	2 (20%)	3 (30%)	5 (50%)	5 (50%)
1	3 (30%)	2 (30%)	3 (30%)	4 (40%)
2	3 (30%)	2 (20%)	1 (10%)	0 (0%)
3+	2 (20%)	3 (30%)	1 (10%)	1 (10%)
**Marital status, *n* (%)**				
Married/partnered	3 (30%)	4 (40%)	9 (90%)	7 (70%)
Single	7 (70%)	6 (60%)	1 (10%)	3 (30%)
**Delivery mode, *n* (%)**				
Vaginal delivery	4 (40%)	6 (60%)	8 (80%)	7 (70%)
Cesarean delivery	6 (70%)	4 (40%)	2 (20%)	3 (30%)
**PTB category, *n* (%)**				
Spontaneous preterm birth	7 (70%)	-	7 (70%)	-
Medically-induced preterm birth	3 (30%)	-	3 (30%)	-

Abbreviations: BMI, body mass index, FTB, full-term birth, PTB, preterm birth. * BMI data were missing for 10 participants. Categories for maternal and fetal characteristics were marked in bold.

**Table 2:** **Sequencing quality control metrics for extracellular vesicle RNA libraries.** Summary of mRNA and miRNA sequencing metrics for plasma-derived extracellular vesicle samples stratified by gestational outcome and race. Reported metrics include total sequencing reads (millions), GC content (%), mRNA alignment rate (%), and miRNA annotation rate. All samples passed quality control and were included in downstream analyses.

Gestation Outcome	Race	Sample ID	mRNA Seq Reads	GC (%)	Aligned (%)	miRNA Seq Reads	Annotated (%)
FTB	Black	FTB-Black-1	47 M	49%	79%	22 M	23%
FTB	Black	FTB-Black-2	42 M	50%	69%	28 M	18%
FTB	Black	FTB-Black-3	96 M	48%	75%	21 M	11%
FTB	Black	FTB-Black-4	111 M	50%	80%	16 M	13%
FTB	Black	FTB-Black-5	78 M	50%	87%	18 M	44%
PTB	Black	PTB-Black-1	30 M	51%	71%	23 M	63%
PTB	Black	PTB-Black-2	34 M	47%	88%	23 M	55%
PTB	Black	PTB-Black-3	86 M	49%	76%	27 M	3%
PTB	Black	PTB-Black-4	87 M	51%	86%	20 M	21%
PTB	Black	PTB-Black-5	84 M	50%	79%	17 M	27%
FTB	White	FTB-White-1	70 M	51%	72%	19 M	9%
FTB	White	FTB-White-2	97 M	50%	82%	15 M	8%
FTB	White	FTB-White-3	96 M	49%	82%	20 M	31%
FTB	White	FTB-White-4	47 M	46%	88%	28 M	51%
FTB	White	FTB-White-5	38 M	48%	86%	30 M	62%
PTB	White	PTB-White-1	86 M	51%	86%	20 M	48%
PTB	White	PTB-White-2	84 M	50%	73%	20 M	18%
PTB	White	PTB-White-3	31 M	45%	88%	26 M	40%
PTB	White	PTB-White-4	112 M	48%	87%	22 M	33%
PTB	White	PTB-White-5	93 M	52%	64%	16 M	6%

Abbreviations: EV, extracellular vesicle; FTB, full-term birth, PTB, preterm birth.

## Data Availability

The datasets underlying the results of this study are included in the article and its Supplementary Files and are available from the corresponding author upon reasonable request.

## Supplementary Materials

The following supporting information can be downloaded at: https://www.mdpi.com/article/10.3390/ijms27114739/s1.
