# Supplementary material for: Circulating Extracellular Vesicles Suggest Race-Associated Transcriptomic Differences in Preterm Birth: A Pilot Study
Source: Int J Mol Sci. 2026 May 25;27(11):4739. doi: 10.3390/ijms27114739 (PMC13257038; doi:10.3390/ijms27114739)
Supplement: Supplementary file 1 [file ijms-27-04739-s001.zip › ijms-4297908-supplementary-r1.pdf]

Supplementary Figures

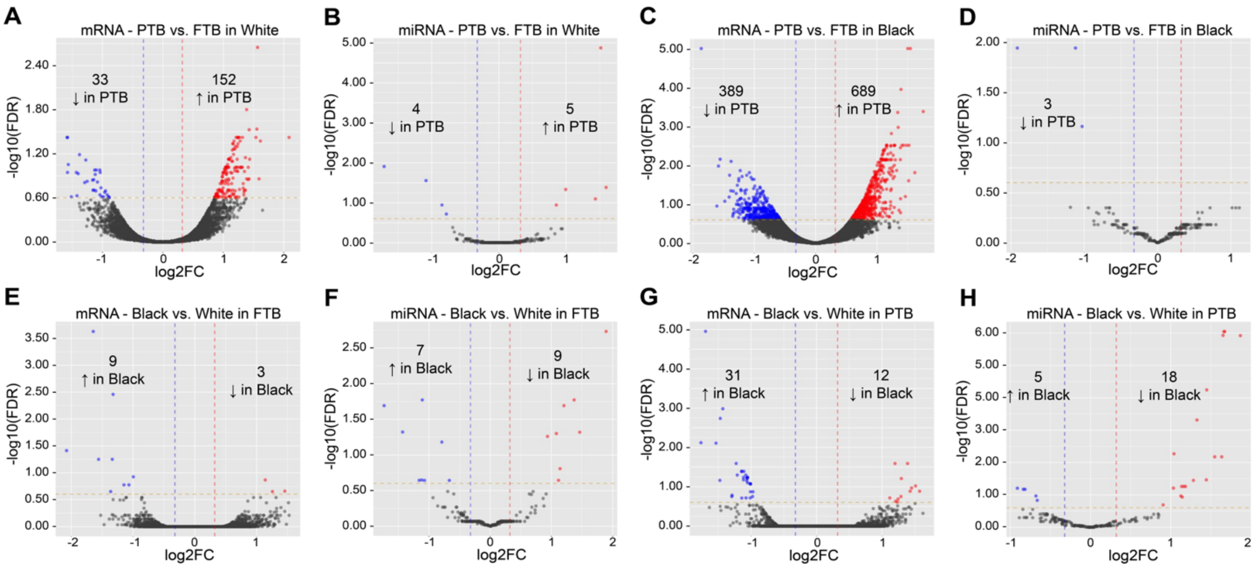

Supplementary Figure S1. Volcano plots of differentially expressed EV RNAs across gestational outcome and race. (A) PTB-White and **(B)** FTB-White differential expression in PTB-White vs FTB-white comparisons. **(C)** EV-mRNA and **(D)** EV-miRNA differential expression in PTB-Black vs. FTB-Black comparisons. (**E**) EV-mRNA and (**F**) EV-miRNA differential expression in FTB-Black vs. FTB-White comparisons. **(G)** EV-mRNA and **(H)** EV-miRNA differential expression in PTB-Black vs PTB-White comparisons. Differential expression was defined as fold change >1.25 and FDR-adjusted q < 0.25. Red points denote transcripts or miRNAs upregulated in PTB or White groups, whereas blue points denote transcripts or miRNAs upregulated in FTB or Black groups, as indicated by each comparison.

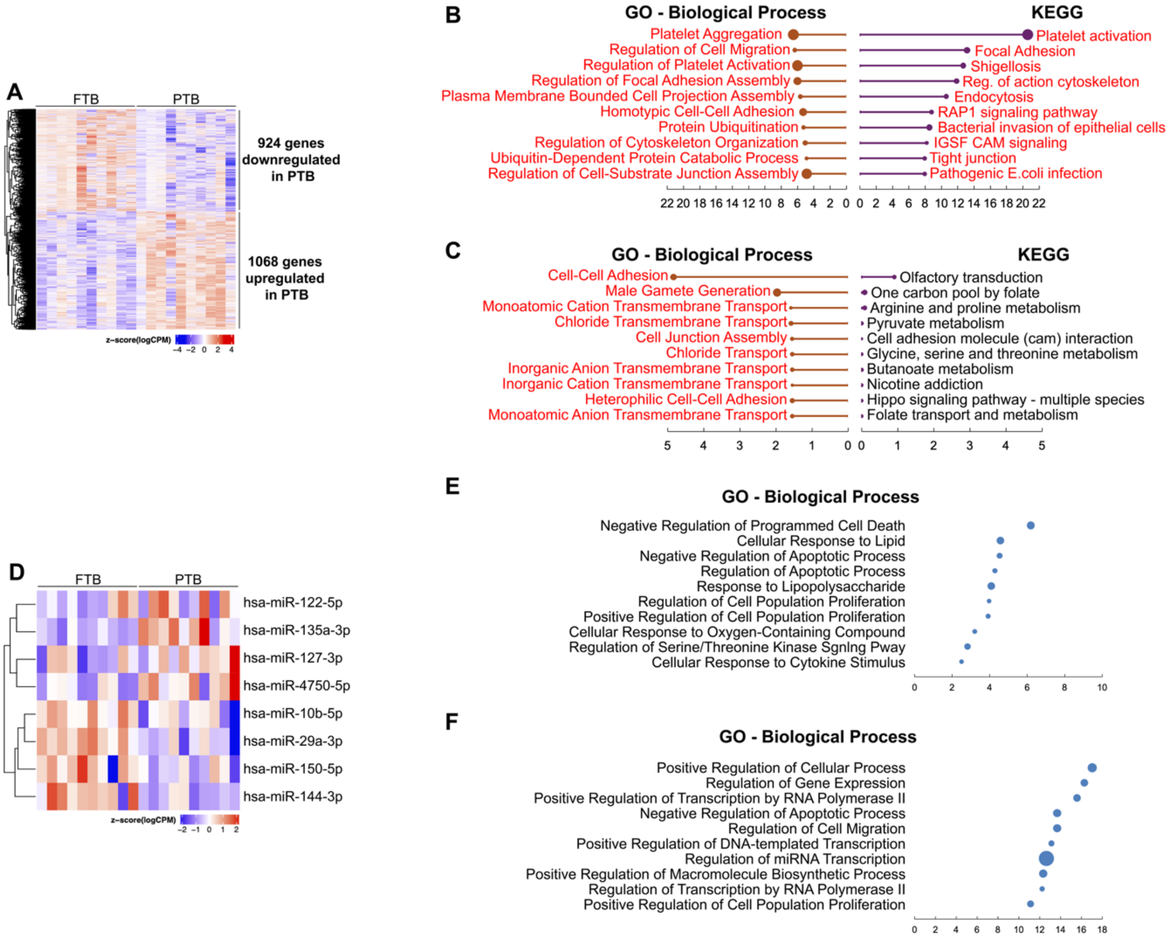

Supplementary Figure S2. Race-independent EV mRNA and miRNA transcriptional signatures associated with PTB. (**A**) Heatmap of differentially expressed EV-associated mRNAs between full-term birth (FTB) and preterm birth (PTB) across the entire study population. Rows represent genes and columns represent individual samples; colors indicate z-score normalized expression levels. (**B**) Enriched Gene Ontology (GO) Biological Process terms and Kyoto Encyclopedia of Genes and Genomes (KEGG) pathways derived from EV-associated mRNAs upregulated in PTB. (**C**) Enriched GO Biological Process terms and KEGG pathways derived from EV-associated mRNAs downregulated in PTB. (**D**) Heatmap of differentially expressed EV-associated miRNAs between FTB and PTB across the entire study population. (**E**) GO Biological Process enrichment analysis of experimentally validated target genes of EV-miRNAs downregulated in PTB. (**F**) GO Biological Process enrichment analysis of experimentally validated target genes of EV-miRNAs upregulated in PTB. Enrichment analyses were performed using Enrichr; pathway significance is shown as −log10(FDR), and dot size represents the combined enrichment score.

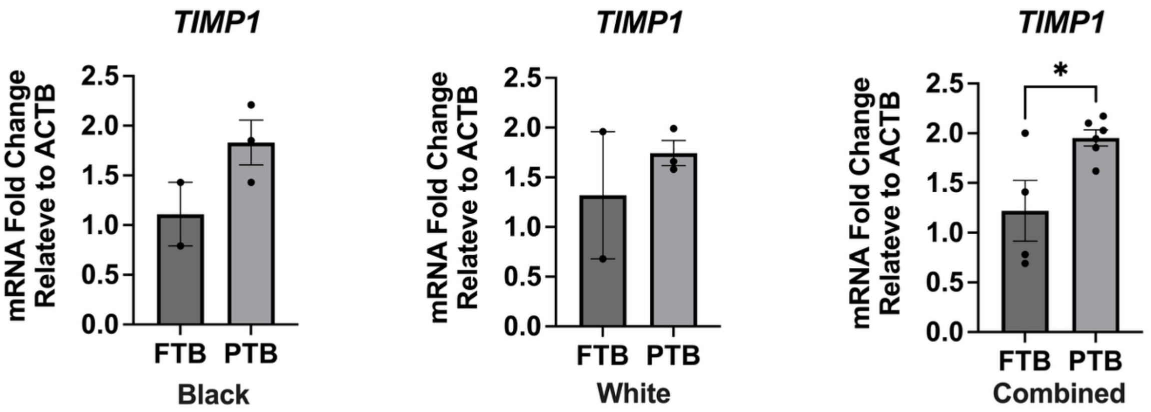

Supplementary Figure S3. *TIMP1* expression changes in PTB EVs compared to FTB EVs. (A) mRNA fold changes of TIMP1 in PTB-Black compared to FTB-Black in relative to housekeeping gene *ACTB*. (**B**) mRNA fold changes of *TIMP1* in PTB-White compared to FTB-White in relative to housekeeping gene ACTB. **(D)** mRNA fold changes of TIMP1 in PTB compared to FTB with Black and White participants combined in relative to housekeeping gene *ACTB*.

Supplementary Table S1. Summary of qRT-PCR validation of selected EV-associated transcripts identified by RNA sequencing. Differentially expressed genes associated with PTB were selected for orthogonal validation by qRT-PCR based on transcriptomic analyses across Black and White participant groups. Reported outcomes indicate whether transcript amplification confirmed RNA-seq directionality, showed inconsistent amplification, or remained below reliable detection thresholds. Technical limitations associated with low-abundance and fragmented EV-associated RNA species may affect amplification efficiency for selected targets.

Gene	RNA-seq finding	RNA-Seq LogFC	RNA-Seq FDR	qRT-PCR outcome
TIMP1	Increased	PTB-Black vs FTB-Black: 1.283	PTB-Black vs FTB-Black: 0.002	Confirmed
		PTB-White vs FTB-White: 1.277	PTB-White vs FTB-White: 0.096	
ENDOD1	Increased	PTB-Black vs FTB-Black: 1.348	PTB-Black vs FTB-Black: 0.001	Below detection
		PTB-White vs FTB-White: 1.555	PTB-White vs FTB-White: 0.029	
ITGA2B	Increased	PTB-Black vs FTB-Black: 1.437	PTB-Black vs FTB-Black: 0.002	Below detection
F2R	Increased	PTB-White vs FTB-White: 1.235	PTB-Black vs FTB-Black: 0.037	Below detection
FCER1G	Increased	PTB-White vs FTB-White: 1.539	PTB-Black vs FTB-Black: 0.037	Below detection
